# Social associations between California sea lions influence the use of a novel foraging ground

**DOI:** 10.1098/rsos.160820

**Published:** 2017-05-17

**Authors:** Zachary A. Schakner, Matthew B. Petelle, Mathew J. Tennis, Bjorn K. Van der Leeuw, Robert T. Stansell, Daniel T. Blumstein

**Affiliations:** 1 Department of Ecology and Evolutionary Biology, University of California, Los Angeles, CA 90095-1606, USA; 2 Department of Zoology and Entomology, University of the Free State, Phuthaditjhaba, South Africa; 3 Pacific States Marine Fisheries Commission, Astoria, OR 97103, USA; 4 Fisheries Field Unit, US Army Corps of Engineers, Cascade Locks, OR 97014, USA

**Keywords:** pinnipeds, native invader, salmonid conservation, human–wildlife conflict

## Abstract

Social relationships define an individual's position in its social network, which can influence the acquisition and spread of information and behavioural variants through the population. Thus, when nuisance behaviours spread through wildlife populations, identifying central individuals may provide valuable insights for problem-species management. We studied the effects of network position on California sea lion (*Zalophus californianus*) discovery and foraging success at a novel foraging ground—the salmonids that aggregate at the Bonneville Dam tail-race, 235 km up the Columbia River. We found that an individual's centrality in their social network influenced discovery of the Bonneville Dam and whether they returned the next year. Foraging success once at the dam was independent of network position. Extensive lethal and non-lethal removal efforts have been implemented at Bonneville Dam and focused on reducing the number of individual sea lions at the dam. Since social relationships forged at the opening of the Columbia River influence both the discovery and return to the Bonneville Dam, efforts to increase salmon recovery may be enhanced by breaking apart social networks at the opening of the river.

## 1. Introduction

Patterns of anthropogenic activity rapidly modify and shift natural food resources, while creating novel resources for animals like croplands, garbage cans, fishing lines and domesticated livestock [1–4]. In a rapidly changing human-dominated environment, animals often cope by shifting foraging strategies to exploit these novel human-derived resources. Under such variable conditions, individual search strategies can be costly, time consuming and risky [5,6]. Social sources of information circumvent these costs by enabling individuals to exploit the knowledge of experienced conspecifics to locate novel foraging grounds, novel prey or acquire novel foraging techniques [7]. Examples of individuals using social information to exploit novel human-derived sources include depredation of fishing lines in marine mammals, and garbage pilfering in black bears (*Ursus americanus*; [8–11]). Benefits of exploiting anthropogenic resources can be high because resources are often concentrated (i.e. fishing lines or crops), but there are potential costs using social information for foraging [12,13].

Costs of relying on social information include the risk of receiving outdated information, especially because competition for foraging resources or territories increases through time [14]. Both population structure and an individual's contact network mediate access to social information [15,16], as information flows non-randomly through populations [17,18]. Thus, an individual's social relationships will influence exposure to the behaviour.

Social network analysis allows us to quantify individual contact patterns to understand how social structure may explain variation in social transmission of foraging patterns. The concept of centrality is relevant to the transmission of behaviours because it captures the relative importance of an individual in the network [17]. When novel foraging information is socially transmitted through a population, the individual's position within its greater social network may therefore influence its foraging behaviour [15]. Less socially embedded individuals may be less privy to information or acquire social information too late, which may have consequences in terms of their foraging success [12–14]. Little is known about how social network structure influences foraging behaviour, but there is growing evidence that an individual's network position can influence discovery of novel resources [15] and therefore its foraging success.

**Figure 1.**
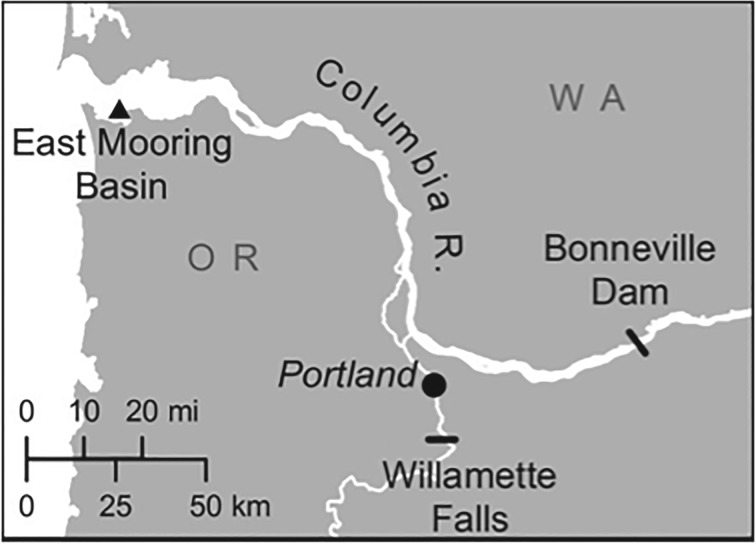
Columbia River with locations of East Mooring Basin (EMB) and Bonneville Dam.

In the Pacific Northwest of the USA, dam construction in salmon spawning rivers create novel concentrations of salmonids that congregate below fish ladders while migrating upriver. In a previous study, we showed that knowledge of a novel food source has been socially transmitted through the population via California sea lion (*Zalophus californianus*) social networks [11]. Specifically, we described the socially mediated increase of sea lions exploiting concentrated salmonids at the Bonneville Dam. The opening of the Columbia River (figure 1) has several major California sea lion haul-out sites, with aggregations of tens to hundreds of migratory males during spring and summer months [19]. Foraging in the Columbia River estuary is not new, but observations of sea lions at the Bonneville Dam (235 km up river—figure 1) from when it was first built until the 1990s were rare [20]. California sea lions, whose populations have grown in a human-dominated environment, are creating invasive species-like effects because of their impacts on salmon populations, some of which are endangered [21]. Not all individuals in Columbia River estuary visit the dam [22], and not all the individuals that have discovered the dam become successful foragers there [20]. Current lethal and non-lethal management activities aim to reduce the number of individual sea lions at the dam, but it is unknown why some individuals have greater predation success than others.

The use of the Bonneville foraging site was socially transmitted through the population, but little is known about how an individual's centrality influences both discovery and predation success at the dam. Here, we used social network analysis to test whether centrality, or the extent to which individual sea lions are embedded within the population network influence foraging at a novel resource; the fish ladders of the Bonneville Dam. While testing for this, we controlled for a variety of other factors including the effects of individual behavioural characteristics (foraging effort and experience), and competition from conspecifics or heterospecifics. Quantifying individual variation in sea lion foraging allows us to understand the mechanisms that create within-population variation in foraging strategies [23]. Our data come from long-term observations of individually identified sea lions' interactions at the opening of the Columbia River with observations of individuals predating salmonids at the Bonneville Dam. We linked foraging success (number of salmon predated) with the population social network to ask whether and, if so, why individuals differed in their foraging success. We had three objectives. First, to identify whether an individual's position within the social network predicts discovery of the dam as well as foraging success in terms of salmon predated. Second, to determine whether there are individual differences in predation after controlling for environmental factors. Third, to understand what effects influence why some individuals become repeat foragers at Bonneville in subsequent seasons.

## 2. Material and methods

### 2.1. Observation effort

#### 2.1.1. East Mooring Basin

California sea lion presence is well documented in the Lower Columbia River, Oregon–Washington. Since 1997, Pacific States Marine Fisheries Commission and Oregon Department of Fish and Wildlife observers have branded, identified and counted the number of individuals at the East Mooring Basin (EMB) (figure 1). We used sight–resight data of branded individuals hauled out at the EMB opening of the Columbia River (figure 1) collected by the Pacific States Marine Fisheries Commission to create an association matrix to calculate associations between pairs of individuals as proportion of times they were seen together. Sampling occurred by performing counts of all individuals hauled out, followed by observing brands at each haul-out. In rare circumstances, when an individual's brand was not visible, observers flushed all animals at the specific haulout and observed brands during rehaul-out. Because individuals rapidly rehauled out and did not flee, this method allowed observers to observe brands that were previously obstructed.

#### 2.1.2. Bonneville Dam

California sea lion predation on salmonids at Bonneville Dam has been monitored by United States Army Corps of Engineers biologists for over a decade [20]. Observations by trained observers during weekday daylight hours at Bonneville Dam began with the first appearance of sea lions at the dam (which varied annually—observer methods in [20]) and continued until their absence. Because sea lions bring large prey items, such as adult salmonids, to the surface during feeding, observers were able to determine their diet [24]. Observers also recorded the number of pinnipeds present, and identified individuals by noting a combination of physical characteristics such as brands, cuts, scars, lumps, colour patterns, size and their estimated age [20]. We used this dataset to determine which individuals from the EMB association matrix data used the Bonneville Dam foraging site, when they were first observed, and to quantify their annual foraging impact on salmon.

### 2.2. Analyses

**Figure 2.**
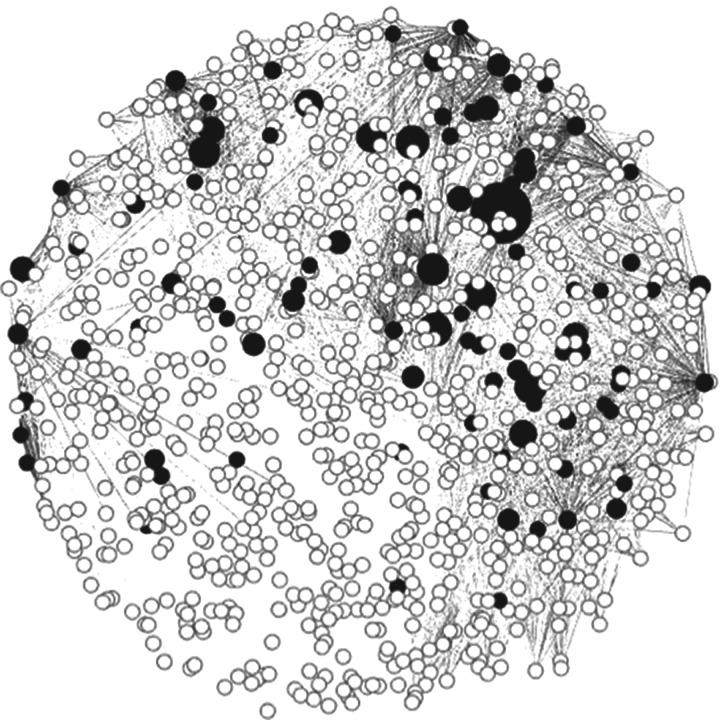
California sea lion social network at the East Mooring Basin, Bonneville foragers and non-Bonneville foragers. Black nodes represent Bonneville foragers; size is scaled to relative salmon consumption.

The social network was created using the simple-ratio association index in SOCPROG [25]. We used a gambit of the group approach [26] with individuals considered associated if they were observed occupying the same dock or jetty area. The association index ranged from 0 (individuals never co-observed) to 1 (two individuals always sighted together). We restricted our analyses to individuals that were sighted at least 10 times (over all years) to accurately estimate the association indices and limit bias from rarely observed individuals. Little is known about male California sea lion social relationships, but males migrate from breeding grounds on the Channel Islands off California. We previously found that individual co-occurrence in the EMB provides a coarse estimate of social ties [11]. For the current analysis, we elected to use the repeated co-occurrence on a haulout within the EMB as a more precise measure of social ties because it measures social relationships over time, and across haul-out locations (figure 2).

We examined differences in foraging behaviour for four social network centrality metrics: betweenness, closeness centrality, clustering coefficient and eigenvector centrality. Centrality metric descriptions are detailed in [27]. Closeness centrality quantifies connectedness of an individual in terms of interactions with every member of the network. Betweenness centrality captures the capacity for a group member to control paths of information between members in a group. Eigenvector centrality measures how much an individual's associates are themselves connected to others in the network. Clustering coefficient quantifies the local density of the subnetwork of a focal individual's neighbours. All social network measures were calculated in R v. 2.14 [28] using the package igraph [29] and the network was displayed using GEPHI [30].

To test whether social network position predicted the use of the novel Bonneville dam foraging site, we fitted a generalized linear model with betweenness, closeness centrality, clustering coefficient and eigenvector centrality as independent variables. The dependent variable was presence/absence at Bonneville Dam and we used a binomial distribution and checked for overdispersion. Since social networks measures are non-independent, multicollinearity was checked using variance inflation factor (VIF) from the AED package [31]. All VIF values were below the threshold of 3 indicating collinearity was not an issue.

To study foraging success (number of salmon taken), we fitted a generalized linear mixed effects model with the total number of salmon predated by an individual for the entire study period as a function of date of first observed arrival, days observed at the dam, whether the individual was ever observed to return to the dam, the mean daily number of California sea lions present at the dam for a given year, whether the cull had been enacted, and the social network node-level characteristics (the same as above) on Bonneville foragers. We fit individual and year as random effects. We scaled all continuous predictor variables (the mean of the variable was subtracted from all individual values) and fitted the model with a Poisson distribution and a square root link to correct for non-normal variance. An observation-level variable was included to account for overdispersion [32]. To assess whether there were individual or year differences in foraging success, we fitted a model without each random effect and performed a log-likelihood ratio test between models with and without the effect. Repeatability was estimated by taking the variance attributed to individual $\left(\sigma_{\alpha }^{2}/(\sigma_{\alpha }^{2}+\sigma_{\varepsilon }^{2}+0.25)\right)$. Where $\sigma_{\alpha }^{2}$ is the variance attributed to individual and $\sigma_{\varepsilon }^{2}$ is the residual variance including year and the observation-level variance.

To study factors that predicted whether an individual returned to the dam the next season, we fitted a generalized linear mixed effects model with a binomial link function using whether or not the individual returned the subsequent year as the dependent variable. Known culled or known dead individuals were removed from the analysis for subsequent years (*n* = 10). Fixed effects included the scaled number of salmon removed from the previous year, the mean daily number of heterospecifics (Steller sea lions *Eumetopias jubatus*), mean daily number of conspecifics and the network centrality metrics on a total of 265 observations. Individual was included as a random effect. The model was fit with a binomial distribution and checked for overdispersion.

For all statistical analyses, we used R v. 2.14 [28] using the package lme4 [33]. We checked residual plots using the DHARMa package. Confidence intervals (CIs) for fixed effects were calculated using the ‘confint’ function (method ‘Wald’) in lme4.

## 3. Results

At the EMB, the social network included 1007 sea lions based upon 51 531 co-occurrences from 1997 to 2014. There were an average of 16 individuals sighted per sampling period (day). Individuals were resighted a mean of 51 times (median of 36, range of 10–391) over the entire study period. Within the social network, 97 individuals used the Bonneville foraging site. The social network statistics did not vary among individuals who did/did not use the dam as a foraging site (electronic supplementary material, figure S2) average residence at Bonneville was 15 days (median = 10, range 0–91) with a mean of 15 salmon predated per season (median = 6, range 0–198). Of the individuals that were sighted at Bonneville, 66 returned after their first season, but the percentage that continues to return declined in subsequent years.

Closeness was positively associated with the use of the Bonneville dam foraging ground (table 1), whereas eigenvector centrality was negatively associated with arriving at Bonneville.

**Table 1.** Results from generalized linear mixed effects model for whether individuals used the Bonneville dam foraging site (binomial response: presence or absence at Bonneville). Model coefficients presented on the scale of the response variable. Significant (*p* < 0.05) variables are given in italics.

variable	estimate	lower 95% CI	upper 95% CI	*p*-value
*intercept*	*0.14*	*0.11*	*0.18*	*<0.001*
*eigenvector centrality*	*0.73*	*0.57*	*0.93*	*<0.05*
*closeness*	*1.61*	*1.11*	*2.50*	*<0.05*
betweenness	0.90	0.64	1.17	0.47
clustering coefficient	1.19	0.88	1.60	0.25

We found that days present at the dam and whether the cull was present were positively associated with foraging success (table 2). The average number of conspecifics had a negative effect on foraging. We did not find, however, an effect of date of first arrival, whether they returned in subsequent years to the dam, or any of the social network metrics on foraging success. Individual was significant and explained 26.48% of the variation (LRT = 12.487; *p* < 0.001); year was also significant (LRT = 6.587; *p* = 0.010).

**Table 2.** Generalized linear mixed effects models for sea lion predation success (*N* salmon predated) at Bonneville Dam. Model coefficients presented on the scale of the response variable. Significant (*p* < 0.05) variables are given in italics.

variable	estimate	lower 95% CI	upper 95% CI	*p*-value
*intercept*	*11.7*	*6.22*	*21.7*	*<0.001*
*cull present*	*3.70*	*1.30*	*10.7*	*0.02*
date first observed	0.97	0.78	1.20	0.69
*days at dam*	*4.78*	*3.75*	*6.09*	*<0.001*
return forager	1.51	0.92	2.49	0.10
*mean daily conspecifics*	*0.65*	*0.45*	*0.92*	*0.02*
closeness	0.99	0.58	1.68	0.95
eigenvector centrality	1.121	0.767	2.49	0.35
betweenness	1.15	0.77	1.72	0.50
clustering coefficient	0.99	0.60	1.65	0.97

Sea lions that were successful in the previous year and had high closeness were more likely to return, but were less likely to return as the number of conspecifics increased (table 3). The number of heterospecifics (Steller sea lions) and betweenness were negatively but not significantly associated with the likelihood of returning (table 3).

**Table 3.** Estimates, standard errors and *p*-values for fixed effects in the generalized linear model for whether an individual California sea lions returned to the Bonneville Dam. Significant (*p* < 0.05) variables are given in italics.

variable	estimate	lower 95% CI	upper 95% CI	*p*-value
*intercept*	*3.17*	*1.94*	*5.18*	*<0.001*
cull present	0.65	0.190	2.216	0.490
*previous year's take*	*1.52*	*1.07*	*2.15*	*0.02*
mean daily heterospecifics	0.50	0.32	1.01	0.05
*mean daily conspecifics*	*0.72*	*0.51*	*0.99*	*0.05*
*closeness*	*2.35*	*1.37*	*4.04*	*0.01*
eigenvector centrality	1.12	0.77	1.64	0.55
betweenness	0.66	0.42	1.05	0.08
clustering coefficient	1.52	0.98	2.36	0.06

## 4. Discussion

We quantified the social networks of individually identified sea lions at the EMB and found that an individual's centrality in their network influenced discovery of the Bonneville Dam and whether they subsequently returned the following season. We previously used network-based diffusion analysis and epidemiological models to demonstrate that the knowledge of the foraging resources at Bonneville was transmitted like a disease [11]. We extended this to show that, like a disease, an individual's network centrality influences the transmission process. Specifically, centrality, captured as closeness, predicted discovery and return to the dam. More central individuals appear to be more ‘susceptible’ to foraging at Bonneville Dam.

The social learning mechanisms by which the foraging information is transmitted are unknown, but we believe simple mechanisms, such as local enhancement, can explain the observed foraging patterns. The haul-out at EMB, the opening of the Columbia river, may serve as an information centre from which unsuccessful foragers can follow successful foragers to the dam. As centrality predicted arrival and return to the dam but not success while at the dam, it appears that individuals may be attracted to the dam by following experienced foragers from the EMB. Once at the dam, we found that predation success was influenced by individual foraging effort (days present at the dam) rather than social factors, suggesting that observational learning from experienced conspecifics on how to forage was not occurring.

Why did one centrality metric predict foraging at Bonneville while others did not? Eigenvector centrality, which measures an individual's connection to more connected individuals, was negatively correlated with predation success. This counterintuitive result may highlight the role of competition, because an individual with more strongly connected affiliates may create competitive pressure. Additionally, some centrality metrics may capture only certain transmission processes. While a single interaction can facilitate transmission of simple contagions, some behaviours require repeated observations or interactions between observer and demonstrator (i.e. complex contagion; [34,35]). For transmission of complex contagion, multiple connections create multiple opportunities for a naive individual to observe experienced individuals. Thus, metrics that capture social influence and redundancy of paths, like the clustering coefficient, may describe the behavioural transmission of complex contagion [35]. In our case, more connected individuals, those that were characterized by having higher closeness, predicted the susceptibility to foraging at the dam, suggesting that simpler contagions are operating.

Dams in salmon spawning rivers create novel concentrations of prey in a confined area. Our results show that when benefits of foraging are high, the effects of competition are magnified. Individuals predate fewer salmon and are less likely to return as the mean daily number of California sea lions present at the dam increases. The increase in competition (both conspecific and heterospecific) may deter individuals from arriving or staying at the dam. Interestingly, the initiation of the sea lion cull was associated with increased salmon predation. This, along with the negative effect of the presence of conspecifics, highlights the role of competition in foraging at novel human-derived resources. It appears that with the initiation of culling in 2008, the successful repeated foragers were lethally removed. But, because the transmission process was well established, new individuals were recruited to the dam and filled the open foraging niches. However, it is important to note that while culling appears to increase relative foraging success, the overall number of salmon predated is still lower after culling.

Even after controlling for competition and individual factors, we found that individuals consistently differed in their foraging success. This suggests some individuals are simply more adept at catching prey than others. Individual differences in body size may be one factor influencing foraging success at Bonneville, but direct measurements of mass were not taken. Larger body size enhances diving ability and foraging efficiency in the open ocean [36]. The effect of size may be enhanced in the shallow tail-race of the Bonneville dam if larger individuals are able to competitively displace smaller individuals at foraging hot spots. Information on branded individuals and weights of animals at the opening of the Columbia River may shed light on whether body size predicts foraging success at the dam. It does not appear, however, that individual learning is a source of variation in foraging success because previous experience at the dam does not explain foraging success. This finding suggests that some individuals arrive at Bonneville as better foragers than others.

Understanding the dynamics of information transmission can not only yield insights into how species adaptively respond to a human-induced changes [37], but are also necessary for management activities aimed at reducing the incidence of problem behaviours [38]. California sea lion range expansion and subsequent predation on threatened and endangered salmonids may create ecological impacts [21]. Predation by small numbers of sea lions drove the winter steelhead (*Oncorhynchus mykiss*) run to critically low levels at Ballard Locks (Seattle, WA, USA), suggesting that even small numbers of individuals exhibit greater *per capita* influence on food webs and population viability [21]. Extensive lethal and non-lethal removal efforts have been implemented at Bonneville Dam and focused on reducing the number of individual sea lions at the dam. Since social relationships forged at the opening of the Columbia River influence both the discovery and return to the Bonneville dam, efforts to increase salmon recovery may be enhanced by breaking apart social networks at the opening of the river. The EMB is an anthropogenic structure that sea lions have co-opted as a popular haul-out. As a result, this area has become a social hub or information reservoir where naive individuals interact with experienced Bonneville foragers. We show that these interactions are central to the recruitment of successful foragers to Bonneville. Management activities that prevent sea lion haul-out at the EMB would inhibit the social interactions that contribute to foraging at Bonneville; simultaneously increasing the efficacy of management interventions while reducing the need for long-term culling.

## Supplementary Material

Electronic Supplementary Material including Study Map, Network statistics, and frequency distributions

## Data accessibility

Our data are deposited at Dryad: http://dx.doi.org/10.5061/dryad.7d80n [39].
