# Supplementary material for: Social associations between California sea lions influence the use of a novel foraging ground
Source: R Soc Open Sci. 2017 May 17;4(5):160820. doi: 10.1098/rsos.160820 (PMC5451787; doi:10.1098/rsos.160820)

**SUPPLEMENTARY MATERIAL:**

**Figure S1 Location of Study Area**


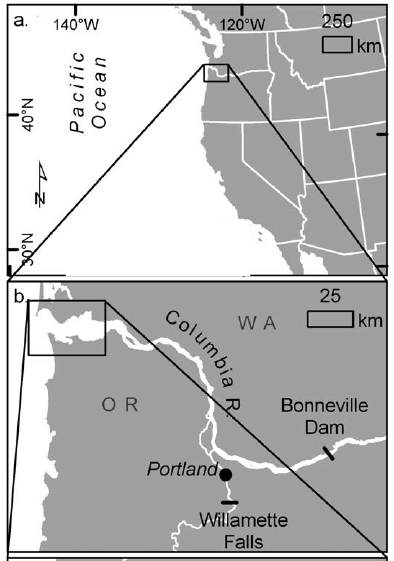


Table S1.

Network centrality measures at the East Mooring Basin and CL_i_ = Closeness, EV_i_ = eigenvector centrality, CC_i_ = clustering coefficient, B_i_ = betweenness.

| I | CL_i_ | EV_i_ | CC_i_ | B_i_ |
| --- | --- | --- | --- | --- |
| Observed | 0.05 | 0.22 | 0.64 | 510 |
| Random | 5.4x10^-4^(5.3x10^-4^, 5.4x10^-4)^ | 0.29 (.28, 0.30) | 0.46 (0.45,0.46) | 85 (81-87) |

We compared the observed average of four centrality measures (closeness, betweeness centrality, eigenvector centrality and clustering coefficient) with the average network measures derived from 1000 randomizations from shuffling edges between nodes. All observed values were significantly different than the expected values (p < 0.05) with CI in parentheses)

Figure S2. Frequency distributions for foragers (green) and non-foragers (yellow) for social network centrality measures.


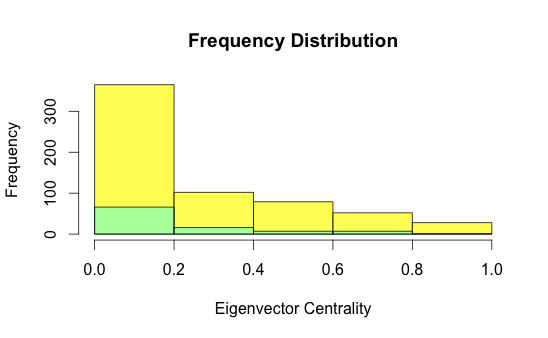

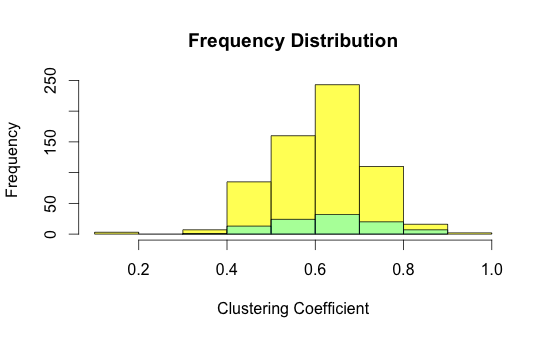

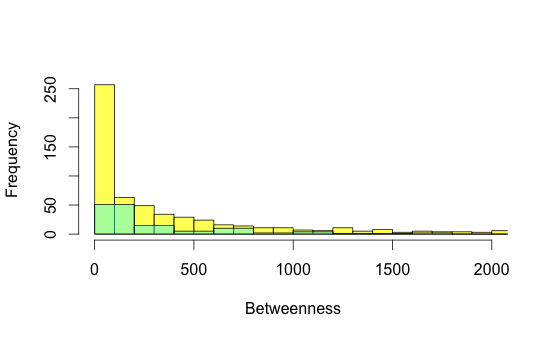


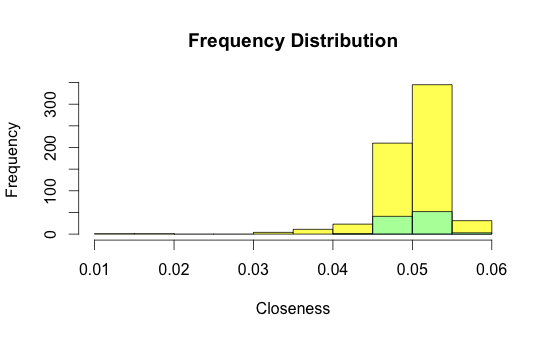

Supplement: Electronic Supplementary Material including Study Map, Network statistics, and frequency distributions [file rsos160820supp1.docx]
